# Papillary Thyroid Carcinoma Arising From a Thyroglossal Duct Cyst: A Case Report

**DOI:** 10.7759/cureus.83982

**Published:** 2025-05-12

**Authors:** Shun Mochida, Gai Yamashita, Kunihiko Tokashiki, Isaku Okamoto, Kiyoaki Tsukahara

**Affiliations:** 1 Otolaryngology - Head and Neck Surgery, Tokyo Medical University Hachioji Medical Center, Tokyo, JPN; 2 Otolaryngology, Nerima General Hospital, Tokyo, JPN; 3 Otolaryngology - Head and Neck Surgery, Tokyo Medical University, Tokyo, JPN

**Keywords:** fine-needle aspiration technology, sistrunk procedure, s: thyroglossal duct cyst, thyroglossal duct carcinomas, thyroid papillary carcinoma

## Abstract

The thyroglossal duct is an epithelial tract that connects the foramen cecum to the thyroid gland and typically regresses during embryonic development. When remnants of this duct persist, they can form cysts known as thyroglossal duct cysts. Although generally benign, these cysts can undergo malignant transformation, most commonly into papillary carcinoma.

Here, we report a case of thyroglossal duct carcinoma in a 22-year-old woman who presented with submental swelling. Imaging revealed features suggestive of malignancy, including microcalcifications and irregular solid components within the cyst. However, multiple fine-needle aspiration cytology (FNAC) procedures failed to detect malignancy. Based on the imaging findings, the patient underwent Sistrunk surgery, and postoperative histopathological analysis confirmed papillary carcinoma arising from a thyroglossal duct cyst.

The diagnosis was challenging due to the tumor's localization within a limited area of the cyst. One year after surgery, the patient showed no signs of recurrence or metastasis. This case highlights the importance of considering malignancy in midline neck masses, even when FNAC results are inconclusive, and underscores the role of early diagnosis and appropriate surgical intervention.

## Introduction

The thyroglossal duct is an epithelial tract that forms during the embryologic descent of the thyroid primordium from the foramen cecum at the base of the tongue to its final position in the neck. Normally, this duct involutes and disappears by the seventh week of gestation. When this process is incomplete, thyroglossal duct remnants may persist and form a cystic structure, known as a thyroglossal duct cyst (TGDC) [1].

TGDCs are common congenital midline neck anomalies and are typically benign. However, malignant transformation occurs in approximately 1-2% of cases [2-3], with papillary thyroid carcinoma (PTC) being the most common histologic type, accounting for approximately 85-90% of TGDC carcinomas (TGDCa), according to a recent systematic review [4]. Although rare, these carcinomas are clinically significant as they pose diagnostic challenges and have the potential for local invasion.

Fine-needle aspiration cytology (FNAC) (a procedure where cells are extracted using a thin needle for microscopic examination) is often employed for preoperative evaluation, but its accuracy in TGDCa is limited. In cystic lesions, aspirated samples frequently consist of acellular fluid or non-representative epithelium, which can lead to false-negative results. This limitation underscores the importance of correlating imaging findings with cytological data when malignancy is suspected.

In this report, we describe a case of PTC arising in a TGDC in a young adult. This case emphasizes the diagnostic limitations of FNAC and the need for careful clinical and radiologic assessments when evaluating midline cystic neck masses.

This article was previously presented as an abstract at the 74th Annual Meeting of the Japan Broncho-Esophagological Society on November 14, 2023.

## Case presentation

**Figure 1:**
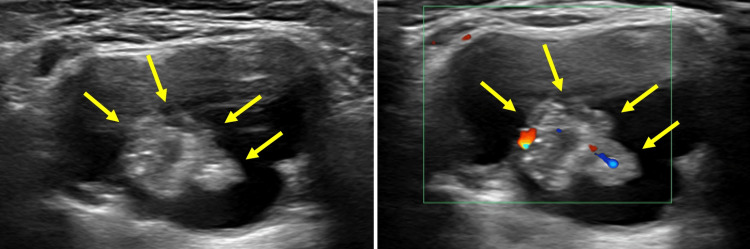
Preoperative ultrasound images A well-defined, lobulated, cystic lesion measuring 45 × 38 × 23 mm is observed in the midline above the hyoid bone. The lesion exhibits internal features suggestive of microcalcifications and sand-like echogenicity.

A 22-year-old woman presented to our hospital with swelling in the submental region. The patient had no significant medical history. The swelling, which was noticed approximately two months prior to presentation, prompted a referral to our hospital for further evaluation and treatment. The swelling was painless, with no associated symptoms such as dysphagia, hoarseness, or fever. Given the absence of specific clinical features, diagnostic evaluation was primarily guided by imaging findings, which raised suspicion of malignancy. On physical examination, a 30-mm mass was palpable in the submental region. The mass was elastic, soft, and poorly mobile. There were no abnormalities observed in the oral cavity, nasal cavity, pharynx, or larynx, and no signs of vocal cord paralysis were present. The patient reported no pain or discomfort at the site of swelling. At the initial presentation, the differential diagnosis included benign congenital lesions such as TGDC, dermoid cyst, and lymphadenopathy. Given the absence of tenderness, rapid growth, or systemic signs, an inflammatory etiology was considered less likely. However, due to the imaging findings suggestive of malignancy, including internal calcifications and solid components, further evaluation was prioritized. Blood tests revealed thyroid function within normal ranges, and the squamous cell carcinoma antigen was also within normal limits (Table 1). Ultrasonography revealed a well-defined, lobulated, cystic lesion measuring 45 × 38 × 23 mm in the midline above the hyoid bone. The volume of the lesion, calculated using the ellipsoid formula (length × width × height × 0.523), was approximately 20.5 cm³, providing insight into the extent of the cystic mass. The lesion exhibited internal features suggestive of microcalcifications and sand-like echogenicity, suggestive of malignancy (Figure 1). The thyroid gland showed no abnormalities, such as nodules, masses, or other suspicious findings.

**Table 1:** Laboratory tests at first visit FT3 - free triiodothyronine; FT4 - free thyroxine; SCC - squamous cell carcinoma antigen; TSH - thyroid-stimulating hormone

Variable	Result	Reference range
TSH	2.38 μIU/mL	0.5-5.0 μIU/mL
FT3	3.81 pg/mL	2.3-4.0 pg/mL
FT4	1.53 ng/dL	0.9-1.7 ng/dL
SCC	0.6 ng/mL	<1.5 ng/mL

**Figure 2:**
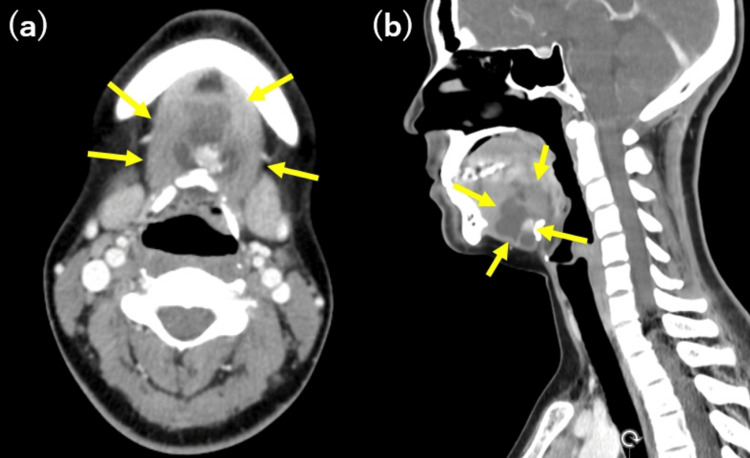
Preoperative contrast-enhanced computed tomography (CT) images (a) Axial view. (b) Sagittal view. A mass is seen anterior to the hyoid bone with internal calcifications. The images are obtained with contrast enhancement. The yellow arrow indicates the cystic component of the lesion.

Computed tomography (CT) revealed a mass anterior to the hyoid bone with internal calcifications (Figure 2).

**Figure 3:**
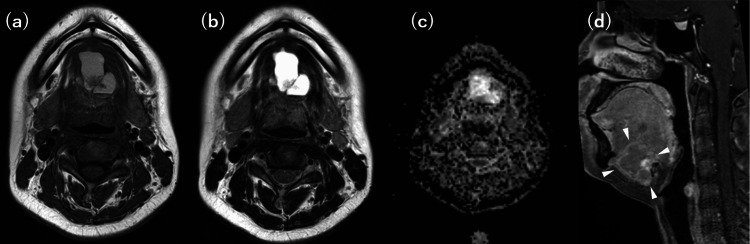
Preoperative contrast-enhanced magnetic resonance imaging (MRI) scans (a) Axial T1-weighted image. (b) Axial T2-weighted image. (c) Apparent diffusion coefficient (ADC) map. (d) Contrast-enhanced sagittal T1-weighted image. The mass demonstrates slightly high signal intensity on T1-weighted images and high signal intensity on T2-weighted images, consistent with a hemorrhagic cyst. The solid components of the mass show contrast enhancement and mild reduction in ADC values, raising suspicion for malignancy.

Contrast-enhanced magnetic resonance imaging (CE-MRI) showed a mass with slightly high signal intensity on T1-weighted images and high signal intensity on T2-weighted images, suggesting a hemorrhagic cyst. The solid components of the mass showed enhancement and mild reduction in apparent diffusion coefficient (ADC) values, raising the suspicion of malignancy (Figure 3).

FNAC was performed on two separate occasions. On the first occasion, FNA was used to collect cystic fluid and smear samples, but the cytological findings were nondiagnostic, showing only benign-appearing cyst contents without atypical cells. On the second occasion, both FNA and core needle biopsy (CNB) were performed, this time targeting the solid component of the lesion. Again, the FNA result remained inconclusive, and CNB suggested a suspicious lesion but did not provide a definitive diagnosis of malignancy. Immunohistochemical analysis of the CNB specimen revealed a Ki-67 labeling index of approximately 35%, with positive staining for cytokeratin AE1/AE3, CK7, and CK20. The sample was negative for cytokeratin 5/6, PAX8, thyroglobulin, TTF-1, and p53, while p40 showed weak focal positivity. These findings were suggestive of carcinoma but not specific to PTC. However, given the concerning imaging findings and inability to exclude malignancy, surgical treatment was planned. Surgery was performed using the Sistrunk procedure. Intraoperatively, the tumor was found to adhere to the hyoid bone, the anterior belly of the digastric muscle, and the mylohyoid muscle, all of which were carefully dissected. The tumor was successfully excised en bloc without rupture.

**Figure 4:**
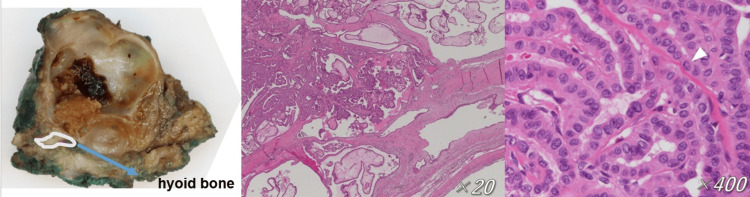
Postoperative pathology of TGDC carcinomas (TGDCa) Histopathological findings of the tumor. Dysmorphic epithelial cells are seen in the full component. The white arrowhead (▽) indicates a tumor cell with an intranuclear cytoplasmic inclusion. The cells show characteristic features of papillary carcinoma, including ground-glass nuclei, nuclear grooves, and intranuclear cytoplasmic inclusions.

Histopathological examination revealed a unilocular cystic lesion measuring 32 mm, located just above the hyoid bone. The cyst contained a 9-mm solid component, which was confirmed to be a papillary carcinoma (Figure 4). The final diagnosis was a TGDCa, with the tumor cells confined within the cyst wall. There was no invasion of the hyoid bone or surrounding muscles. The surgical margins were negative. No vascular or neural invasion was observed, either histologically or intraoperatively. The patient’s postoperative recovery was uneventful, and she was discharged on postoperative day 7. No recurrence or metastasis was observed during the one-year follow-up period.

## Discussion

The thyroglossal duct forms during the descent of the thyroid primordium from the foramen cecum to its final anatomical location. The involution takes place by the seventh to 10th week of embryogenesis. Failure of involution, combined with the presence of residual thyroid tissue, may result in the formation of TGDC [5]. Approximately 98% of TGDCs are benign; however, thyroid tissue is present in up to 80% of cases [2]. Among TGDCa, approximately 90% are reported to be papillary carcinomas. The risk of malignancy arising from thyroid tissue in TGDCs is approximately 1.6-1.9%, which is three to 16 times higher than the reported prevalence of thyroid cancer in the general population (0.12-0.63%), according to Ojiri [6]. The histological findings in the present case were consistent with those of PTC. Differential diagnoses of midline neck masses include dermoid cysts, branchial cleft cysts, and reactive lymphadenopathy. These entities are typically benign and often lack concerning imaging features such as internal microcalcifications, irregular or solid components, or enhancement on contrast imaging. In contrast, the present case exhibited multiple radiologic features suggestive of malignancy, including microcalcifications, enhancement of the solid component, and reduced ADC values on MRI. These findings helped distinguish this lesion from more common benign midline neck masses and supported the decision to perform surgery.

Ultrasonography is the primary diagnostic tool for evaluating thyroid nodules, including TGDCs. It has reported sensitivity and specificity rates of 68.18% and 76.55%, respectively, for detecting thyroid nodules [7]. Although other imaging modalities, CT, MRI, and fluorodeoxyglucose positron emission tomography (FDG-PET), are useful for staging the extent of disease, their role in qualitative assessment is limited [8]. Malignant ultrasound findings include hypoechogenicity, irregular or microlobulated margins, microcalcifications, taller-than-wide shape, intramodular vascularity, and extrathyroidal extension. In the current case, ultrasonography and CT findings, including microcalcification and internal echogenicity, raised concerns about malignancy, which was confirmed through histopathological examination following surgical excision. These malignant ultrasound findings are indicative of TGDCa [9]. These features highlight the importance of careful imaging assessment in identifying potential malignancies in TGDCs. However, ultrasonography has limitations in detecting malignancy within TGDCs, particularly when the lesion is deeply located, predominantly cystic, or has a very small solid component. In such cases, CT and MRI may provide complementary information, including the identification of calcifications, evaluation of soft tissue invasion, and more accurate lesion characterization. Additionally, FDG-PET can assess the metabolic activity of the lesion and may aid in the diagnosis and detection of metastasis or recurrence. Therefore, when ultrasound findings are inconclusive or suggest malignancy, a multimodal imaging strategy that includes CT, MRI, and FDG-PET should be considered.

FNAC is commonly used to evaluate thyroid lesions, with a reported sensitivity of 89.31%, specificity of 48.44%, and diagnostic accuracy of 75.89% for thyroid nodules [4]. However, FNAC of cystic components, such as TGDCa, often has a lower diagnostic yield, with an accuracy rate as low as 20.6% [4]. Several factors contribute to this variability in diagnostic accuracy. These include the cystic nature of the lesion, which limits the presence and accessibility of malignant cells, and the tendency for FNAC to sample only cystic fluid or residual thyroid tissue rather than representative tumor cells. In the present case, the solid component was relatively small (9 mm), deeply located near the hyoid bone, and partly embedded within the cyst wall. These anatomical and structural factors likely reduced the likelihood of obtaining adequate cellular material and contributed to the false-negative results. Additionally, variations in operator technique, needle placement accuracy, and interpretation of cytological samples may also influence FNAC accuracy. This diagnostic limitation emphasizes the need for a high index of suspicion based on imaging findings, such as microcalcification or irregular borders, especially when FNAC results are inconclusive. Even when FNAC results are negative, malignancy should be considered, and appropriate surgical intervention should be planned. In our case, immunohistochemical staining of the CNB specimen yielded findings consistent with malignancy but inconclusive for PTC, as thyroglobulin, TTF-1, and PAX8 were negative. Although cytokeratin markers (AE1/AE3, CK7, CK20) were positive and Ki-67 showed a high proliferation index, these features did not lead to a definitive preoperative diagnosis. Furthermore, thyroglobulin levels in aspirated cystic fluid were not measured, and no study clearly supports its utility in diagnosing TGDCa, although it has been discussed as a potential adjunctive marker.

The prognosis of TGDCa is generally excellent, with a 10-year survival rate approaching 100% [10] and a cure rate of 95% [11] following the Sistrunk operation. When malignancy is suspected, the standard surgical approach is the Sistrunk procedure, which includes en bloc resection of the cyst, the entire thyroglossal duct tract, and the central portion of the hyoid bone. The Sistrunk procedure remains the gold standard for TGDC management. In cases with cervical lymph node metastasis, neck dissection should be added. Total thyroidectomy may also be considered to enable postoperative radioactive iodine ablation. Studies have shown no significant survival difference between patients treated with the Sistrunk operation alone and those undergoing additional thyroidectomy. Prophylactic neck dissection is not recommended unless there is clear evidence of lymph node metastasis. Recurrence rates for TGDCa are low (4.3%), with the cervical lymph nodes being the most common site of recurrence (42.9%) [4]. Therefore, close follow-up is essential to monitor for any sign of recurrence or metastasis, although the overall risk remains low with appropriate surgical management. Recurrence most commonly occurs at the primary site and in the cervical lymph nodes, and follow-up with neck ultrasonography and CT is recommended. According to a systematic review, total thyroidectomy and radioactive iodine therapy may be considered in selected cases of TGDCa, particularly in patients aged >45 years or in the presence of aggressive features such as lymph node involvement or extracapsular extension. However, routine thyroidectomy is not recommended in all cases, as the incidence of concomitant thyroid malignancy is relatively low (approximately 23.4%), and the risks associated with thyroidectomy must be weighed against the potential benefits [4]. Although this report is based on a single case, it highlights diagnostic limitations in current cytological techniques. Further studies with larger case series are needed to refine diagnostic strategies for TGDCa, especially in cystic lesions.

Specifically, future research should explore the use of CNB, thyroglobulin measurement in aspirated cyst fluid, and advanced imaging modalities such as diffusion-weighted MRI or FDG-PET to improve preoperative diagnosis. In addition, large-scale, multicenter investigations may contribute to the development of standardized diagnostic and treatment protocols for TGDCa.

## Conclusions

We report a case of PTC arising from TGDC without a definitive preoperative diagnosis. Although TGDCs are generally benign, their potential for malignancy must be considered during diagnosis and treatment planning. Clinicians should be aware of the possibility of malignancy in cases of TGDC, particularly when clinical and imaging findings are atypical. In this case, despite repeated preoperative evaluations including imaging and biopsy, a definitive diagnosis could not be established until surgery. This highlights the diagnostic challenges associated with TGDC malignancy and underscores the importance of considering surgical intervention when malignancy cannot be ruled out. This case emphasizes the need for heightened awareness and further research on the malignancy potential of TGDCs.
